# Supplementary material for: Opioid Prescription Method for Breathlessness Due to Non-Cancer Chronic Respiratory Diseases: A Systematic Review
Source: Int J Environ Res Public Health. 2022 Apr 18;19(8):4907. doi: 10.3390/ijerph19084907 (PMC9024433; doi:10.3390/ijerph19084907)
Supplement: Supplementary file 1 [file ijerph-19-04907-s001.zip › ijerph-1625352-supplementary.pdf]

Table S1. Research strategy in MEDLINE and Chochrane

MEDLINE

	Search formula
L1	S RESPIRATORY INSUFFICIENCY+NT/CT OR LUNG DISEASES, INTERSTITIAL+NT/CT OR PULMONARY DISEASE, CHRONIC OBSTRUCTIVE+NT/CT OR BRONCHIECTASIS+NT/CT
L2	S RESPIRATORY(3A)(INSUFFICIEN? OR FAILUR?) OR INTERSTIT?(3A)(LUNG OR PULMONARY OR PNEUMON?) OR CHRONIC?(3A)OBSTRUCT?(3A)(LUNG OR PULMONARY OR AIRWAY OR AIRFLOW?)
L3	S BRONCHIECTAS? OR FIBROS?(3A)(PULMONARY OR ALVEOLI?) OR EMPHYSEM?(3A)(PULMONARY OR FOCAL OR PANACINAR? OR PANLOBUL? OR CENTRIACINAR?) OR CHRONIC?(3A)BRONCHIT? OR ((CYSTIC FIBROSIS+NT/CT OR CYSTIC?(3A)FIBROS?) AND (LUNG OR PULMONARY))
L4	S (REFRACTORY? OR INTRACTABL?)(3A)(DYSPNE? OR DYSPNOE? OR BREATHLESS? OR BREATH(3A)SHORT?) OR *DYSPNEA+NT/CT OR (DYSPNE? OR DYSPNOE? OR BREATHLESS? OR BREATH?(3A)SHORT?)/TI
L5	S ANALGESICS, OPIOID+NT/CT OR MORPHINE DERIVATIVES+NT/CT OR FENTANYL+NT/CT OR METHADONE+NT/CT OR TRAMADOL+NT/CT OR BUPRENORPHINE+NT/CT
L6	S OPIOID? OR MORPHIN? OR OXYCODON? OR ?FENTANYL? OR METHADON? OR TRAMADOL? OR BUPRENORPHIN? OR ?CODEIN? OR HYDROMORPHON?
L7	S (L1-L4) AND (L5 OR L6)
L8	S END(2W)LIFE OR PALLIATIVE CARE+NT/CT OR TERMINAL CARE+NT/CT OR TERMINAL? OR PALLIAT? OR SEVERE? OR ADVANCED?
L9	S NONCANCER? OR (NON OR WITHOUT)(W)CANCER?
L10	S L8 AND L9
L11	S L8 NOT (NEOPLASMS+NT/CT OR (CANCER? OR CARCINOM? OR TUMOR? OR TUMOUR? OR NEOPLAS? OR MALIGNAN? OR ONCOLOG?))
L12	S L7 AND (L10 OR L11)
L13	S L7 AND L6/TI AND (DYSPNE? OR DYSPNOE? OR BREATHLESSNES? OR BREATH?(3A)SHORT? OR BRONCHI? OR FIBROS? OR EMPHYSEM? OR RESPIRATORY(3A)(FAILUR? OR INSUFFICIEN?) OR INTERSTIT? OR OBSTRUCT? OR COPD OR COAD OR IP OR ILD)/TI
L14	S L13 AND L9
L15	S L13 NOT (NEOPLASMS+NT/CT OR (CANCER? OR CARCINOM? OR TUMOR? OR TUMOUR? OR NEOPLAS? OR MALIGNAN? OR ONCOLOG?))
L16	S L14 OR L15
L17	S L12 OR L16
L18	S L17 AND (1980-2020)/PY AND (19800101-20201031)/UP AND (ENGLISH OR JAPANESE)/LA NOT EPUB?/FS

Chochrane

	Search formula
#1	[mh "RESPIRATORY INSUFFICIENCY"] OR [mh "LUNG DISEASES, INTERSTITIAL"] OR [mh "PULMONARY DISEASE, CHRONIC OBSTRUCTIVE"] OR [mh BRONCHIECTASIS]
#2	(RESPIRATORY near/2 (INSUFFICIENC* OR FAILURE*) OR INTERSTITIAL near/3 (LUNG OR PULMONARY OR PNEUMONI*) OR CHRONIC near/3 OBSTRUCT* near/3 (LUNG OR PULMONARY OR AIRWAY OR AIRFLOW*)):ti,kw,ab
#3	(BRONCHIECTAS* OR FIBROSIS near/3 (PULMONARY OR ALVEOLIT*) OR EMPHYSEMA near/3 (PULMONARY OR FOCAL OR PANACINAR OR PANLOBUAR OR CENTRIACINAR) OR CHRONIC near/3 BRONCHITI*):ti,kw,ab
#4	([mh "CYSTIC FIBROSIS"] OR CYSTIC near/3 FIBROSIS:ti,kw,ab) AND (LUNG OR PULMONARY):ti,kw,ab
#5	((REFRACTORY OR INTRACTABLE) near/3 (DYSPNE* OR DYSPNOE* OR BREATHLESS* OR BREATH near/3 SHORT*)):ti,kw,ab OR [mh DYSPNEA[mj]] OR (DYSPNE* OR DYSPNOE* OR BREATHLESS* OR BREATH near/3 SHORT*):ti
#6	#1 or #2 or #3 or #4 or #5
#7	[mh "ANALGESICS, OPIOID"] OR [mh "MORPHINE DERIVATIVES"] OR [mh FENTANYL] OR [mh METHADONE] OR [mh TRAMADOL] OR [mh BUPRENORPHINE]
#8	(OPIOID* OR MORPHIN* OR OXYCODON* OR *FENTANYL* OR METHADON* OR TRAMADOL* OR BUPRENORPHIN* OR *CODEIN* OR HYDROMORPHON*):ti,kw,ab
#9	#6 and (#7 or #8)
#10	([mh "PALLIATIVE CARE"] OR [mh "TERMINAL CARE"] OR (END near/2 LIFE OR TERMINAL* OR PALLIAT* OR SEVERE* OR ADVANCED*):ti,kw,ab)
#11	NONCANCER* OR (NON OR WITHOUT) next CANCER*:ti,kw,ab
#12	#10 and #11
#13	#10 NOT ([mh NEOPLASMS] OR (CANCER* OR CARCINOM* OR TUMOR* OR TUMOUR* OR NEOPLAS* OR MALIGNAN* OR ONCOLOG*):ti,kw,ab)
#14	#9 and (#12 or #13)
#15	#9 and (DYSPNE* OR DYSPNOE* OR BREATHLESS* OR BRONCHI* OR FIBROS* OR EMPHYSEM* OR FAILUR* OR INSUFFICIEN* OR INTERSTIT* OR OBSTRUCT* OR COPD OR COAD OR IP OR ILD):ti and (OPIOID* OR MORPHIN* OR OXYCODON* OR *FENTANYL* OR METHADON* OR TRAMADOL* OR BUPRENORPHIN* OR *CODEIN* OR HYDROMORPHON*):ti
#16	#15 and #11
#17	#15 NOT ([mh NEOPLASMS] OR (CANCER* OR CARCINOM* OR TUMOR* OR TUMOUR* OR NEOPLAS* OR MALIGNAN* OR ONCOLOG*):ti,kw,ab)
#18	#14 or #16 or #17
#19	#18 with Cochrane Library publication date Between Jan 1980 and Oct 2020, in Cochrane Reviews, Cochrane Protocols, Clinical Answers, Editorials, Special Collections
#20	#18 with Publication Year from 1980 to 2020, in Trials
#21	#19 or #20

Table S2. Bias risk of the randomized controlled studies.

Study	Random sequence generation (Selection bias)	Allocation concealment (Selection bias)	Blinding of participants and personnel (performance bias)	Binding of outcomes assessmnet (detection bias)	Incomplete outcome data (attrition bias)	Selective reporting (reporting bias)	Other bias
Verberkt CA, (2020)[11]	Low risk	Unclear risk	Low risk	Unclear risk	Low risk	Low risk	Low risk
Currow D, (2020)[12]	Low risk	Low risk	Low risk	Unclear risk	Low risk	Low risk	Low risk
Kronborg-White S, (2020)[29]	Low risk	Unclear risk	Low risk	Unclear risk	Low risk	Low risk	Low risk
Ferreira DH, (2019)[21]	Low risk	Low risk	Low risk	Unclear risk	Low risk	Low risk	Low risk
Abdallah SJ, (2017)[30]	Unclear risk	Unclear risk	Low risk	Unclear risk	High risk	Low risk	Low risk
Abernethy AP, (2003)[25]	Low risk	Low risk	Unclear risk	Unclear risk	Low risk	Low risk	Low risk
Poole PJ, (1998)[31]	Low risk	Unclear risk	Unclear risk	Unclear risk	High risk	Low risk	Low risk
Light RW, (1996)[19]	Unclear risk	Unclear risk	Low risk	Unclear risk	Low risk	Low risk	Low risk
Eiser N, (1991)[32] 1	Unclear risk	Unclear risk	Unclear risk	Unclear risk	High risk	Low risk	Low risk
Eiser N, (1991)[32] 2	Unclear risk	Unclear risk	Low risk	Unclear risk	Low risk	Low risk	Low risk
Munck LK, (1990)[33]	Unclear risk	Unclear risk	Low risk	Unclear risk	Low risk	Low risk	Low risk
Light RW, (1989)[34]	Unclear risk	Unclear risk	High risk	Unclear risk	Low risk	Low risk	Low risk
Rice KL, (1987)[35]	Unclear risk	Unclear risk	Low risk	Unclear risk	High risk	Low risk	Low risk

Johnson MA, (1983)[36]	Unclear risk	Unclear risk	Unclear risk	Unclear risk	Low risk	Low risk	Low risk
Woodcock AA, (1982)[18]	Unclear risk	Unclear risk	Unclear risk	Unclear risk	High risk	Low risk	Low risk
Woodcock AA, (1981)[37]	Unclear risk	Unclear risk	Unclear risk	Unclear risk	Low risk	Low risk	Low risk

Table S3. Bias risk of non-randomized studies, observational studies, five retrospective studies.

Study	Selection of participants (Selection bias)	Confounding variables (Selection bias)	Measurement of exposure (Performance bias)	Blinding of outcome assessments	Incomplete outcome data	Selective outcome reporting
Rocker GM, (2013)[38]	High risk	Low risk	Low risk	Unclear risk	Low risk	Low risk
Currow DC, (2011) [26]	Low risk	Low risk	Low risk	Unclear risk	Low risk	Low risk
Allcroft P, (2013)[39]	High risk	Unclear risk	Low risk	Unclear risk	Low risk	Low risk
Allen S, (2005) [40]	High risk	Low risk	Low risk	Unclear risk	Low risk	Low risk
Smallwood N, (2018)[41]	Low risk	Low risk	Low risk	Unclear risk	Low risk	Low risk
Vicent L, (2017) [42]	Low risk	Low risk	Low risk	Unclear risk	Low risk	Low risk
Takeyasu M, (2016)[43]	High risk	Low risk	Low risk	Unclear risk	Low risk	Low risk
Matsuda Y, (2017) [44]	High risk	Low risk	Low risk	Unclear risk	Low risk	Low risk
Bajwah S, (2012)[5]	High risk	Low risk	Low risk	Unclear risk	Low risk	Low risk
Tsukuura H, (2013) [45]	High risk	Low risk	Low risk	Unclear risk	Low risk	Low risk
Colman R, (2015) [46]	High risk	Low risk	Low risk	Unclear risk	Low risk	Low risk
